# Biostimulant Potential of *Scenedesmus obliquus* Grown in Brewery Wastewater

**DOI:** 10.3390/molecules25030664

**Published:** 2020-02-04

**Authors:** Elvira Navarro-López, Angela Ruíz-Nieto, Alice Ferreira, F. Gabriel Acién, Luisa Gouveia

**Affiliations:** 1 Department of Chemical Engineering, University of Almería, Cañada San Urbano, s/n, 04120 Almería, Spain; nle877@ual.es (E.N.-L.); angelaruiznieto@gmail.com (A.R.-N.); facien@ual.es (F.G.A.); 2 LNEG, National Laboratory of Energy and Geology I.P., Bioenergy Unit, Estrada do Paço do Lumiar 22, 1649-038 Lisbon, Portugal; alice.ferreira@lneg.pt

**Keywords:** wastewater treatment, *Scenedesmus obliquus*, microalga, biostimulants, germination index, root development

## Abstract

Microalgae are microorganisms with the capacity to contribute to the sustainable and healthy food production, in addition to wastewater treatment. The subject of this work was to determine the potential of *Scenedesmus obliquus* microalga grown in brewery wastewater to act as a plant biostimulant. The germination index of watercress seeds, as well as the auxin-like activity in mung bean and cucumber, and in the cytokinin-like activity in cucumber bioassays were used to evaluate the biostimulant potential. Several biomass processes were studied, such as centrifugation, ultrasonication and enzymatic hydrolysis, as well as the final concentration of microalgal extracts to determine their influence in the biostimulant activity of the *Scenedesmus* biomass. The results showed an increase of 40% on the germination index when using the biomass at 0.1 g/L, without any pre-treatment. For auxin-like activity, the best results (up to 60% with respect to control) were obtained at 0.5 g/L of biomass extract, after a combination of cell disruption, enzymatic hydrolysis and centrifugation. For cytokinin-like activity, the best results (up to 187.5% with respect to control) were achieved without cell disruption, after enzymatic hydrolysis and centrifugation at a biomass extract concentration of 2 g/L.

## 1. Introduction

Wastewater remediation by microalgae is widely described as an efficient nutrient removal, cost-effective and sustainable process, which avoids secondary pollution by using the biomass produced for different applications such as biofuels, bioplastics, biofertilizers and including animal feed and aquaculture [1,2,3,4,5,6,7]. This strategy is a win–win process that benefits both parts because (i) wastewater treatment with microalgae has lower energy demand, GHG emissions and costs than conventional systems, whereas (ii) the utilization of wastewater as nutrients source allows the reduction of microalgae biomass production cost below 5 €/kg [6,8]. To minimize the microalgae biomass production cost, it is mandatory to use open reactors. Moreover, it has been previously reported that minimum values below 1 €/kg are possible when coupling with wastewater treatment and optimizing productivity and harvesting methodology [6].

The utilization of algal-bacterial consortia in wastewater treatment is based on the solar-driven oxygenation by the microalgae thus providing the required oxygen for bacteria to degrade the organic matter, the assimilation of nutrients into the biomass, and the efficient removal of pathogens due to high UV radiation and dissolved oxygen concentrations achieved by these systems [9,10]. Microalgae based wastewater treatment systems are simpler and more energy efficient, requiring less than 0.2 kWh/m^3^ when compared to conventional processes (0.5 kWh/m^3^), thus attracting the attention of companies involved in wastewater treatment [6,11]. These systems have been proposed as useful to solve important problems such as scarcity of water and water pollution, while also contributing to the enhancement of healthy and sustainable food production if microalgae biomass is used to improve the sustainability of farming activities.

Food production through agriculture has been continuously increasing in order to feed the growing world population. However, the current practices have followed a tendency of intensification, relying mainly on the use of chemical fertilizers. The majority of these fertilizers are based in finite mineral resources, and can be toxic, damaging the biosphere and causing various human diseases through food and water contamination [12,13].

Currently, consumers are increasingly appreciating the production of high quality, healthy fruits and vegetables, especially when they are obtained with minimal impact on the environment [14]. New approaches, such as the application of natural products in agriculture, have been proposed not only to improve the productivity and quality of crops and crop-derived products, but also increase the sustainability of agricultural production, avoiding further environmental degradation (e.g., eutrophication, soil infertility and biodiversity loss) [15,16].

Biostimulants are materials, other than fertilizers, that, when applied in small quantities, promote the growth and quality of food crops/vegetables/fruits, boost mineral nutrient uptake and expand tolerance of plants to abiotic stresses. Moreover, they do not generate chemical residues and fully respect the human health and the environment, which makes then a sustainable alternative to synthetic plant protection products [17,18,19,20].

Among biostimulants, a special attention has been given to algae biomass or extracts [21,22] as they contain many groups of active compounds including peptides and amino acids as well as plant growth-promoting substances, e.g., plant hormones (cytokinins, auxins, gibberellins, abscisic acid and ethylene) [18,23,24]. The plant growth induced by biostimulants can be associated with an increase of amino acids and enhanced protein biosynthesis [25]. According to Górka et al. (2016) [26], the algae hormones seem to be the most essential for enhancing the growth and development of crop plants. The microalgae-based biostimulants have high efficiency, full biodegradability, no phytotoxicity, making them promising products for plant production [27]. They can enhance seed germination, improve plant growth, yield, flower set and fruit production, as well as a post-harvest shelf life [16,18,28]. Examples of commercially available algal based products for agriculture have already been reported [21]. However, most of them are obtained from macroalgae, with only a few reports based on microalgae.

According to Regulation (EC) No 2003/2003 (Fertilizers Regulation) there are no restrictions on the utilization of materials from wastewaters for biofertilizer products. However, the obtained products should comply with the rules outlined on this document regarding their minimum nutrient content, their safety and the absence of adverse effects on the environment, allowing it to circulate freely on the European market [29].

One great challenge of microalgae-based biostimulants is the water and nutrients required for microalgae growth, which hinder its viability and sustainability. Thus, this work consisted on the assessment of the biostimulant potential of the microalga *Scenedesmus obliquus* produced using brewery wastewater, avoiding the need of adding clean water and nutrients. *Scenedesmus obliquus* grew very well on the brewery wastewater, while simultaneously allowing an efficient removal of nutrients, which generated a clean water with values below the legislation [3,4]. The biostimulant activity of the obtained biomass was evaluated through different bioassays, including the determination of germination index (gibberellin-like effect), root induction (auxin-like effect) and expansion (cytokinin-like effect) of different crops (watercress, cucumber and mung bean). Furthermore, the influence of the downstream processing (cell disruption, enzymatic hydrolysis and centrifugation) was studied. 

## 2. Results and Discussion

### 2.1. Protein Content and Enzymatic Hydrolysis

First, the protein content of the biomass was measured since amino acids have been reported as highly relevant in the biostimulant effect on plants [30]. To be more effective the proteins must be released as amino acids, either through chemical or enzymatic hydrolysis. Enzymatic hydrolysis, however, is preferred because according to Romero García et al. (2012) [31], the process is more effective and the L-amino acids are better preserved. The protein content of the *Scenedesmus obliquus* biomass grown in brewery effluent was 35.9%. The enzymatic hydrolysis was not greatly affected by the cell disruption using ultrasounds, achieving a hydrolysis degree of 65% against 57%, without any rupture. These values are quite similar with the ones found by Romero García et al. (2012) [31] for *Scenedesmus almeriensis*, who obtained a hydrolysis degree of about 60%.

### 2.2. Biostimulant Activity 

**Figure 1:**
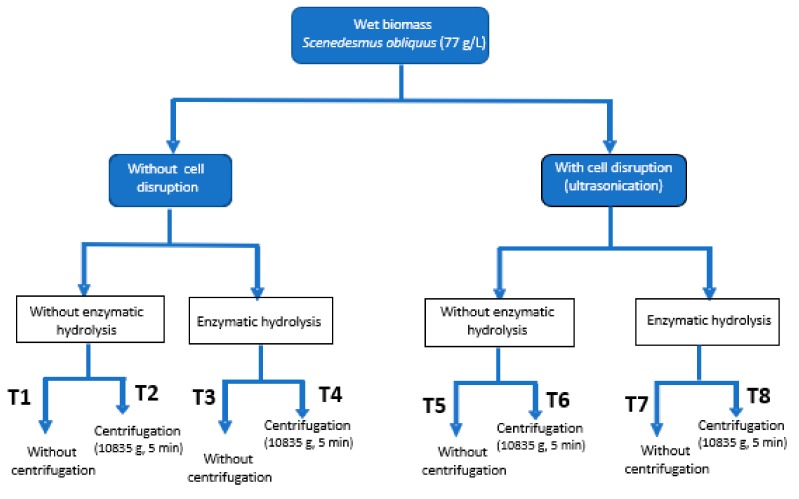
Downstream processing of the wet microalgal biomass (cell rupture by ultrasounds, enzymatic hydrolysis and centrifugation). For each treatment different concentrations were tested (0.1, 0.5 and 2 g/L depending on the bioassay).

To evaluate the biostimulant activity of extracts or products from microalgae two approaches can be performed: complete chemical analysis of elemental composition, amino-acids content and profile and phytohormones content. Alternatively, different bioassays can be performed to evaluate the improvement of plant growth when providing such products. The second strategy is much more practical, although, due to the different biostimulants effects, only one bioassay is not enough. Thus, on this work up to four different methodologies were essayed to identify three main effects: (i) germination index of watercress seeds (gibberellin-like effect), (ii) evaluation of the auxin-effect of microalgae extracts by using mung bean and cucumber seeds and (iii) determination of the cytokinin-like effect with cucumber seeds (Table 1), whereas Table 2 provides a statistical analysis of the above mentioned factors. These four bioassays were done with eight extracts (T1–T8) obtained from the fresh microalgal biomass of *Scenedesmus obliquus* by applying different downstream processing strategies (ultrasounds, enzymatic hydrolysis (EH) and centrifugation) and extract concentration (Figure 1).

#### 2.2.1. Gibberellin-Like Effect. Germination Index

To determine the germination index (GI) it is necessary to take into account that a GI of 100% is attributed to the distilled water (control) and, therefore, only algae extracts that led to values higher than 100% are considered to have biostimulant activity. The highest GI was achieved with the treatment 1 (T1), which corresponds to the initial microalgal biomass, without the application of any pre-treatment, neither cell disruption, enzymatic hydrolysis nor centrifugation, and at the lowest concentration tested, 0.1 g/L (Table 1). In this condition, it was observed a GI of 139.1%, which is almost 40% higher than that obtained with distilled water (control). This result highlights the potential of *Scenedesmus obliquus* grown in brewery effluent as a biostimulant for agriculture applications. Moreover, when compared with the application of a commercial biofertilizer product that leads to GI of 93.1% and 102.1% at 0.1 and 0.5 g/L respectively, the results obtained were much higher. According to the supplier’s instructions, the commercial product should be used at 2 g/L, but in the GI tests and in order to compare with the microalgae extracts, the commercial product was diluted to 0.1 and 0.5 g/L.

These results could demonstrate the presence of molecules with biological activity such as gibberellins, which are a group of plant hormones that play an important role in the initiation of seed germination and stem elongation [32,33]. As it is shown in Table 1, the higher germination indexes of watercress seeds were obtained for treatments without hydrolyzing the biomass, that is T1 and T2 (undisrupted biomass) and T5 and T6 (disrupted biomass). The use of the hydrolyzed biomass as raw material (T3, T4, T7 and T8), germination indexes below 100% were obtained in all cases, which demonstrated a certain inhibitory effect on the germination of the seeds when the hydrolysate from the microalgal biomass were used as substrate.

The enzymatic hydrolysis was performed in order to evaluate the potential of the hydrolysates from *Scenedesmus obliquus* as raw material for the production of biostimulants as this treatment increases the concentration of amino acids and polyamines directly related with the plant growth development. These biomolecules are in charge of the initiation of defensive responses and of growth regulation at micromolar concentrations, respectively [32]. As it was mentioned before, higher germination rates were obtained in the case of not hydrolyzing the microalgae biomass, whether or not the biomass has been previously broken by ultrasound. This result could be due to the fact that the undisrupted biomass has already an optimal concentration of amino acids and/or polyamines as it can be seen that lower concentrations give the best efficacy on the stimulant effect. Subjecting the biomass to any pre-treatment, such as cell disruption or enzymatic hydrolysis, could increase the concentration of both compounds leading to an inhibition of the seeds growth. This inhibitory effect observed on seed growth with the increase of the extract concentration was already described on *Triticum aestivum* var. Pusa Gold, and it could be due to the presence of some plant regulators that caused an inhibitory effect on the growth of the watercress seeds when these compounds exceed a certain optimal concentration [34]. It is well known that the effect of algal extract on the plant growth depends on factors, such as dose, method and time of application, and selected cultivar [35]. According to Davies, (2004) [36] and Tarakhovskaya et al. (2007) [32] special attention should be paid to the phytohormones, which are bioactive compounds that influence physiological process in plants at low concentrations and can inhibit at higher concentration. Only a few research works have been published about the use of microalgae as biostimulant products for agriculture, sometimes using different methodologies. For example, Ennis et al. (2017) [37] calculated the germination index of cress seed in contact with hydrochars from the microalga *Phaeodactylum tricornutum* to evaluate the influence of fatty acids. The authors achieved a GI of 73% and 102%, respectively for delipidated and non-delipidated hydrochar. The later value increased to 126% when the sample was previously milled.

As it can be observed in Table 1, in most cases, the extract centrifugation had no significant effect on the germination index of watercress seeds (for instance, GI of 118.9% ± 3.2% and 108.2% ± 4.5% were obtained for T1 and T2 at 0.5 g/L without and with centrifugation, respectively). This fact can be verified when the statistical analysis was performed in order to determine which variables mostly affected the germination percentage of the seeds (Table 2). It means that the supernatants of the microalgal extracts could be used as biostimulant products. This factor is important to increase the profitability of the process, as the supernatants obtained after the centrifugation could then be used as a biostimulant product for agricultural application, while the remaining biomass could be used as raw material for the extraction of other bioproducts of interest such as fatty acids, proteins or carotenoids, among others.

Moreover, as it can be observed from Table 2, the main factor affecting the germination index of the seeds is the enzymatic hydrolysis, although there is also a significant effect from the interaction extract concentration–enzymatic hydrolysis.

#### 2.2.2. Auxin-like Effect. Root Formation of Mung Bean and Cucumber

**Figure 2:**
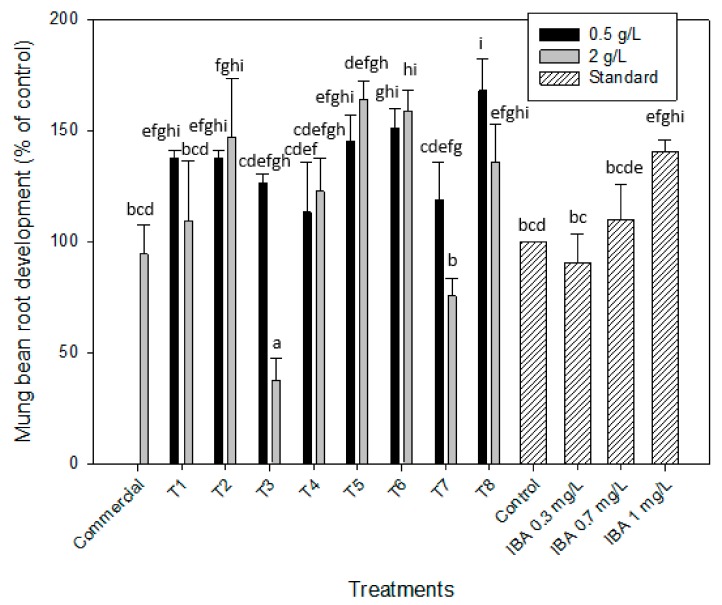
Auxin-like activity of *Scenedesmus obliquus* biomass when applied in the mung bean root development bioassay for the eight microalgal treatments (T1–T8) at the two concentration tested (0.5 and 2 g/L) using indole-3-acetic acid (IBA) at 0.3, 0.7 and 1 mg/L as the auxin standard. Results are shown as mean ± SE (*n* = 3). Different letters indicate significant differences (*p* < 0.05) within each treatment.

Amin et al. (2009) [38] found that the *Spirulina* aqueous extract contained relatively high content of phytohormones such as indole-3-acetic acid (IAA), gibberellic acid (GA), benzyladenine (BA), abscisic acid (ABA), jasmonic acid (JA) and methyl jasmonic acid (MeJA). This statement led the authors to perform the evaluation of the auxins on the induction of the roots in mung bean (*Vigna radiata* L.) and cucumber cotyledons (*Cucumis sativus*). Auxins play a crucial role in the induction of root initiation and in the elongation growth, so this protocol was applied in order to determine the auxin-like activity of the microalgal extracts from *Scenedesmus obliquus*. As it can be seen in Table 1, most of the extracts without enzymatic hydrolysis, as shown in Figure 1 (T1, T2, T5 and T6), led to a greater number of roots in the cotyledons of the mung bean in comparison with the control (distilled water), which would prove the presence of auxins in these samples. Results obtained from this bioassay revealed that the higher effect, 167.9% ± 14.2% was obtained for the pre-treatment T8, that is, for the samples obtained with cell disruption, enzymatic hydrolysis and centrifugation at 0.5 g/L (Figure 2). Nevertheless, this percentage of the root development was quite similar to those obtained for other treatments, such as T5 (with cell disruption, with enzymatic hydrolysis and without centrifugation) at 2 g/L (164.2% ± 8.1%), T6 (with cell disruption, without enzymatic hydrolysis and with centrifugation) at 0.5 and 2 g/L, which led to percentages of 150.9% ± 8.7% and 158.5% ± 9.8%, respectively or T2 (without cell disruption, without enzymatic hydrolysis and with centrifugation) at 2 g/L (147.2% ± 15.5%). The latter (T2) is especially interesting as it represents much less energy and time consuming, turning the process more sustainable. Moreover, the root development obtained with the application of the above treatments are similar to those obtained with the auxin standard at the highest concentration tested, 1 mg/L, as it is demonstrated in Figure 2 with the statistical analysis.

Figure 2 shows the mung bean root development, expressed as a percentage calculated with respect to the control (distilled water), for the eight treatments applied in this work and compared to the auxin indole-3-acetic acid (IBA) standard curve. As it can be observed, only extracts obtained by the treatment T3 (without cell disruption, with EH and without centrifugation) at 2 g/L, did not promote growth (root development of 37.7% ± 7.7%) while others as those obtained with treatment T7 (with cell disruption, with EH and without centrifugation) at 2 g/L led to a root development of 75.5% ± 7.8%, which was not significantly different when compared with the control (distilled water) or the auxin at 0.3 mg/L. These algae extracts obtained by enzymatic hydrolysis at the highest concentration did not show auxin-like activity and it was quite similar to the control, while the same extracts diluted to 0.5 g/L led to a root development of 126.4% ± 4.3% and 118.9% ± 16.9%, respectively for T3 and T7. A similar effect was reached when T1 was applied to the microalgal biomass at the highest concentration (2 g/L; 109.4% ± 26.7%), while at 0.5 g/L this parameter increased to 137.7% ± 3.3%. In all these cases the root development significantly increased when extracts were diluted to 0.5 g/L.

For both cases, better results were obtained at the lowest concentrations, with or without hydrolysis, showing again the possible inhibitory effect on the root development of the mung bean cotyledons. This inhibitory effect by the highest concentration was also confirmed when performing the statistical analysis of the obtained data. As it is shown in Table 2, the one parameter that had the most significant effect (*p*-value < 0.05) on the root development of mung bean was the concentration at which algae extracts were diluted, 0.5 and 2 g/L. In any case, these results highlight the growth promotion capacity of the microalgal biomass of *Scenedesmus obliquus* grown in brewery effluent at early stages of the mung bean plant development. 

Some authors, such as Niemann and Dörffling (1980) [39] have also described that some bioactive compounds, like abscisic acid (ABA) produce shoot growth suppression when they act on their own or form an inhibitory complex with other biomolecules such as lunularic acids and other unidentified compounds, avoiding the plant growth in the bioassays. This fact could explain the inhibitory effect at higher concentrations (2 g/L). Plaza et al. (2018) [33] analyzed the hormone content of the hydrolyzed of *Scenedesmus* sp. and *Arthrospira* sp. and determined an ABA content of 3718.3 and 1.03 ng/g in both microalgae respectively, among others phytohormones such as gibberellins or salicylic acid.

Authors such as Stirk et al. (2020) [40] determined the auxin-like activity of different microalgae extracts from *Chlorella* sp., *Chlorella vulgaris* and *Scenedesmus acutus* obtained after different cell disruption methods by applying a mung bean bioassay [41] different from the one applied in this work. The authors determined that some method of cell disruption is necessary to increase the extraction yield of the bioactive molecules for these microalgae species as sonication or ball mill. As it is shown in Figure 2, a similar result was obtained in this work for the specie *Scenedesmus obliquus* when the mung bean root bioassay was applied as a slight increase of the root development was obtained when samples were firstly ultrasonicated (Treatments 5 to 8). Bumandalai and Tserennadmid (2019) also evaluated the effect of *Chlorella vulgaris* on the growth of tomato and cucumber seeds. They pointed out the increase of 80.8% and 100% on cucumber and tomato roots, respectively, when compared to the control (after 9 days) [42].

**Figure 3:**
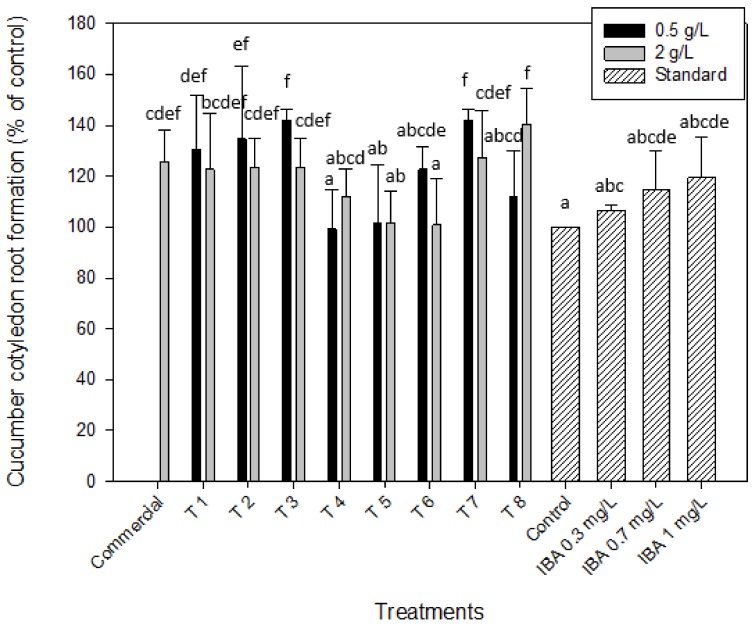
Auxin-like activity of *Scenedesmus obliquus* biomass when applied the excised cucumber cotyledon root formation bioassay for the eight microalgal treatments (T1–T8) at the two concentration tested (0.5 and 2 g/L) and using indole-3-acetic acid (IBA) at 0.3, 0.7 and 1 mg/L as the auxin standard. Results are shown as mean ± SE (*n* = 4). Different letters indicate significant differences (*p* < 0.05) within each treatment.

The excised cucumber cotyledon root formation test was used in order to have another test to confirm the presence of auxins in the microalgal extracts. As it was stated before, auxins play an important role in the initiation of root formation and induction of elongation growth [32]. As it is shown in Figure 3, several extracts obtained from different treatments significantly increased the root formation in comparison with the control, highlighting the effect of the presence of auxins in *Scenedesmus* extracts. The best results were achieved for the biomass after the treatment by enzymatic hydrolysis with or without cell disruption (T7 and T3, respectively) obtaining both a percentage of root development of 141.9% ± 4.6% in comparison with the distilled water (control) at the lowest concentration tested (0.5 g/L). Similar results were obtained with T8 (with cell disruption, enzymatic hydrolysis and centrifugation) at 2 g/L (140.3% ± 14.3%). So, as it can be observed in Figure 3, the root formation obtained with these three treatments highlighted, significantly increased the results obtained when the auxin standard was used at 1 mg/L. Lower percentages of root development were observed on the cucumber cotyledons when more concentrated extracts were used in the same treatments (127.4% ± 18.3% and 123.4% ± 11.6% for T7 and T3 at 2 g/L, respectively). Again, it was observed that for the non-centrifuged extracts, such as T3 and T7, the higher the extracts concentration, and therefore, of the biomolecules responsible of growth, the lower the root development obtained was. These values were at the same magnitude to the one obtained with the commercial product from microalgae, which led to a root development of 125.8% ± 12.1% when it was applied at 2 g/L on the cucumber cotyledons. Similar results were obtained with the microalgal biomass without any treatment (T1) leading to a root development of 130.6% ± 21.3%. 

These results confirm the potential of *Scenedesmus obliquus* as a biostimulant product since in any of the three bioassays applied in this work it was possible to demonstrate the presence of gibberellins (germination index) and auxins (root formation of mung bean and cucumber). Furthermore, the fact that the use of microalgal biomass directly, without any treatment (T1), at the lowest concentrations, reached very positive results was very interesting for making the process less energy intensive and resulting in lower costs and greater profitability of the product.

#### 2.2.3. Cytokinin-like Effect. Cucumber Cotyledon Root Expansion

**Figure 4:**
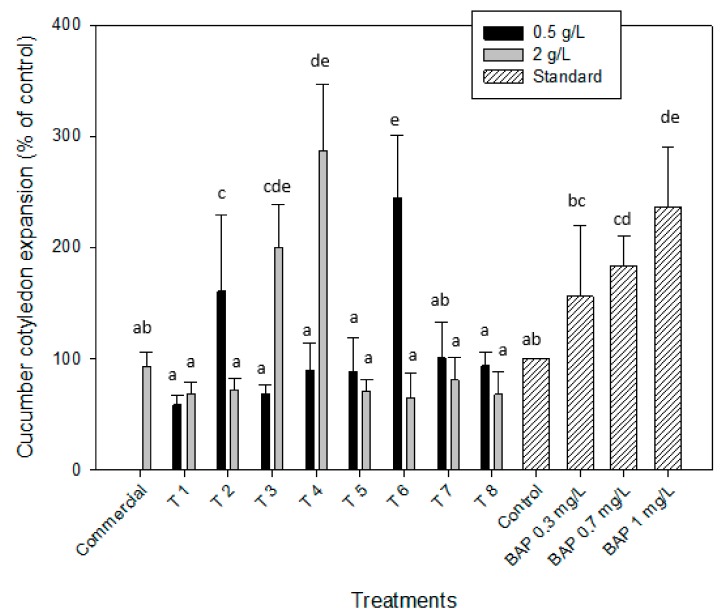
Cytokinin-like activity of *Scenedesmus obliquus* biomass when applying the excised cucumber cotyledon root expansion bioassay for the eight microalgal treatments (T1–T8) at the two concentration tested (0.5 and 2 g/L) and using BAP at 0.3, 0.7 and 1 mg/L as the cytokinin standard. Results are shown as mean ± SE (*n* = 3). Different letters indicate significant differences (*p* < 0.05) within each treatment.

The cucumber cotyledon root expansion test was performed to determine the cytokinin-like activity of the microalgal extracts obtained again from the different downstream processing (T1–T8). Cytokinins are a group of phytohormones that are supposed to be in control of cell division, bud development or development of the leaf blade [32]. Figure 4 depicts the results of the different downstream processing (T1–T8) for the two extract concentrations and compared with the standard curve of the cytokinin 6-benzylaminopurine (BAP), which was tested at three different concentrations 0.3, 0.7 and 1 mg/L. The microalgal extracts from *Scenedesmus obliquus* grown in brewery wastewater pre-treated by T4 (without cell disruption, with EH and after centrifugation at 2 g/L) and T6 (with cell disruption, without EH and after centrifugation at 0.5 g/L) led to the highest percentages of cotyledon expansion, 287.5% ± 59.2% and 245.1% ± 55.9%, respectively. These values were similar to those obtained by applying the cytokinin BAP at a concentration of 1 mg/L (236.1% ± 54.2%) directly on the cucumber cotyledons and it was significantly higher than the distilled water (control) and the commercial biofertilizer made from algae, which led to a cotyledon expansion of 92.9% ± 13.1%. In addition, the extracts subjected to T2 at 0.5 g/L and T3 at 2 g/L allowed expansion percentages higher than the control (160% ± 68.7% and 200.3% ± 38.8%, respectively). The remaining cases were all below, which means they did not present cytokinin-like activity. Stirk et al. (2002) [43] applied this bioassay to evaluate the eventual presence of cytokinins in ten different strains of microalgae and claimed a positive result in all of them, being especially significant in species such as *Scenedesmus quadricuada* and *Chlamydomonas* sp. Furthermore, Barone et al (2019) developed two systems of co-cultivation with tomato seedlings using one microalga specie (*Scenedesmus quadricuada* (Sq) or *Chlorella vulgaris* (Cv)). Sq allowed an increase of 107% and Cv, of 144% of root fresh weight, after 46 days of cultivation [44]. 

## 3. Material and Methods

### 3.1. Microalgae, Enzymes and Chemicals

*Scenedesmus obliquus* (ACOI 204/07) used in this work was from the ACOI Coimbra University Collection of Algae, Portugal. The microalga was grown using brewery wastewater (BWW) from the Sociedade Central de Cervejas e Bebidas (SCC) brewery at Vialonga, Portugal (38° 52′ 59″ N, 9° 3′ 34″ W). This BWW has been pre-treated in a full-scale anaerobic reactor BIOPAQ®IC at the factory’s industrial wastewater treatment plant. The composition of this wastewater is presented in Table 3. The fresh biomass used was the one obtained from the brewery effluent treatment and was already applied in biofuel production trials and was already published elsewhere [3,4,5].

The enzymes used in the enzymatic hydrolysis were Alcalase 2.5 L (2.5 AU-A/g) and Flavourizyme 1000 L (1000 AU-A/g), both of them supplied by Novozymes A/S (Bagsvaerd, Denmark). 

The chemicals used for analytical methods were sodium tetraborate decahydrate, DL-dithiothreitol, phtaldialdehyde, dodecyl sulfate and DL-serine all of them obtained from Sigma-Aldrich (St Louis, MO, USA). All reagents used in the analytical determination were of analytical grade. Standards were purchased from Sigma-Aldrich (St Louis, MO, USA) and used without further purification.

### 3.2. Microalgae Cultivation

The microalga cultivation was done as described by Ferreira et al. (2019) [5]. The photobioreactors (PBRs) used were cylindrical bubble column, with 14 cm diameter and 40 cm height. They were operated in batch mode with a working volume of 4 L. Agitation was provided through air injection at a flow rate of 0.1 vvm (L L^−1^ min^−1^). The cultures were maintained at room temperature (23–25 °C) under constant fluorescent light (43.2 μmol·m^−2^·s^−1^). Cultivation was maintained until the cultures reached stationary phase according to optical density measurements at 540 nm.

### 3.3. Downstream Processing of Microalgal Biomass

**Figure 5:**
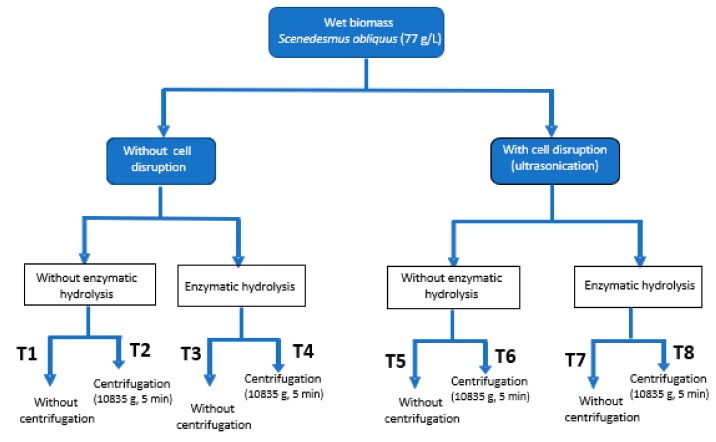
Downstream processing of the fresh microalgal biomass (cell rupture by ultrasounds, enzymatic hydrolysis and centrifugation). For each treatment different concentrations were tested (0.1, 0.5 and 2 g/L depending on the bioassay).

The influence of different downstream processing strategies into the bioactivity of the microalgal biomass was studied. A complete factorial experimental design was performed considering three main steps: cell disruption by ultrasounds, enzymatic hydrolysis at previously optimized conditions, and centrifugation of the resulting sludge to remove waste biomass from the product (Figure 5). Treatments are numbered from T1, which is the initial biomass without any pre-treatment, until T8, which corresponds to the biomass on which the three pre-treatments have been applied, such as cell disruption, enzymatic hydrolysis and centrifugation.

The fresh microalgal biomass from *Scenedesmus obliquus* with an initial concentration of 77 g/L was first subjected to a cell disruption pre-treatment by ultrasonication. After that, both the disrupted and the undisrupted biomass were used as raw material of another treatment by enzymatic hydrolysis, and finally, the effect of the centrifugation of the extracts thus obtained was studied. The microalgal extracts obtained by each way of treatment were diluted to 0.1–2 g/L depending on the bioassay (0.1 and 0.5 g/L for germination index, and 0.5 and 2 g/L for the rest of bioassays carried out in this work).

### 3.4. Cell Disruption by Ultrasounds

For cell disruption the sample was subject to ultrasounds. For that, the microalgae sample of 100 mL was placed in a 500 mL beaker, with a 0.6 kW ultrasonic processor (UP 400S, Hielsher Ultrasonics, Germany) operating at 24 kHz with a flux cell FC22K, allowing the material to be sonicated in continuous flow mode; the ultrasonic processor has variable power output control, which was set at 70% during experiments for 40 min. The sample passed through tubes in the central cylinder where the energy input was released, and then returned to the beaker through a second tube. A vortex was used to guarantee the homogenization of the sample.

### 3.5. Enzymatic Hydrolysis

The enzymatic hydrolysis was conducted in a 100 mL Erlenmeyer flask heated and stirred (300 rpm) with a heating magnetic stirrer (Multimix Heat D, Ovan, Spain) under a temperature of 50 °C and controlled pH [31]. The hydrolysis was carried out in two steps, each at the optimal conditions recommended by the enzyme’s suppliers. In the first step, 50 mL of the microalgal biomass with a concentration of 40 g/L were transferred into an Erlenmeyer flask and heated to 50 °C. The pH was adjusted to 8 by addition of NaOH 1 M. Then, 4% (w/w) of Alcalase 2.5 L was added into the reactor and the reaction began. Free amino acids were released as the reaction proceeded, which acidified the medium leading to a decrease of the pH, which was compensated by adding NaOH 1 M to maintain the pH at 8. After two hours of the beginning of the reaction, pH was lowered to 7 by adding H_2_SO_4_ 1 M and then the second enzyme, Flavourizyme 1000 L was added to the reaction medium. After three hours, it was considered that the reaction was finished, and the solution was heated to 75 °C for 15 min to deactivate the enzymes.

### 3.6. Centrifugation

The resulting sludge of the hydrolysis process was centrifuged in a batch mode at 10,835× *g* for 5 min (Micro-centrifuge Minispin®plus Eppendorf, Hamburg, Germany).

### 3.7. Protein Content and Hydrolysis Degree

The protein content was determined following the method described by González López et al. (2010) [45], which is a modification of the Lowry method, with BSA (Bovine serum albumin) as the standard. The hydrolysis degree is defined as the ratio between the number of free amino acids of each sample and the total number of amino acids-protein available for hydrolysis. The hydrolysis degree was determined using the OPA (o-phthaldialdehyde) method using serine as standard assuming the following value of the parameters of the model α = 1, β = 0.4 and yhtot = 8 [46].

### 3.8. Bioassays to Determine the Biostimulant Potential of the Microalgae Biomass

#### 3.8.1. Germination Index of Watercress seeds (*Lepidium sativum*) for Gibberellins-like Effect

The biostimulant activity of the microalgal extracts was determined by measuring the germination index of *Lepidium sativum,* L. seeds according to the method described by Zucconi et al. (1981) [47]. So, for each treatment, 50 mL of the homogenized biomass solution with an initial concentration of 77 g/L of dry biomass with different downstream processing was transferred to Erlenmeyer flasks of 100 mL and incubated at 30 °C for 2 h with magnetic stirrer at 300 rpm in a heating magnetic stirrer (Multimix Heat D, Ovan, Spain). Resulting extracts were collected and diluted to 0.5 and 0.1 g/L to carry out bioassays in order to quantify the germination index of the seeds in contact with each extract. The germination index bioassay was tested with extracts at concentrations below 1 g/L because on previous experiences it was demonstrated that, for most of microalgae samples, this concentration exerted a toxic effect leading to an important reduction of the germination index.

In each experiment, 100 tested of commercial watercress seeds (*Lepidium sativum*, L., Sonnentor; 25 seeds × 4 petri dishes) were placed on Whatman No 5 filter papers in four sterilized 90 mm Petri dishes and then treated with 2 mL of distilled water (control) and the extracts resulting of each pre-treatment (T1–T8). Finally, the germination index of each sample was determined by the Equation (1), where G and L are the number of germinated seeds and their length in the case of the microalgal extracts and Gw and Lw are the same parameters but in the control (distilled water). The data shown in the germination index experiments is, therefore, the result of the measurement of 100 seeds for each treatment.

(1) $$GI\left(\%\right)=\frac{G\times L}{G_{W}\times L_{W}}\times 100$$

#### 3.8.2. Adventitious Mung Bean (*Vigna radiata*) Root Induction for Auxin-like Effect

Commercial mung bean *Vigna radiata* (L.) Wilczek seeds were planted according to Zhao et al. (1992) [48]. Seeds were planted at 1 cm depth in moistened perlite in plastic trials maintained at 27 °C and illuminated with fluorescent lamps in cycles of 12 h of light in a grown chamber. After 7 days of incubation at these conditions, five seedlings were then cut and placed in vials. Each condition was tested in triplicate so three vials (15 samples) were needed per treatment. After the incubation period in the same conditions described above for seeds growth, the number of roots (longer than 1 mm) were counted on each hypocotyl. The number is directly proportional to the auxin concentration within the assay range. The mean number of roots, derived from each vial, was compared to the controls (water, negative control and commercial product, positive control) and evaluated using a standard curve for comparison made by a specific auxin (Indol-3-butyric acid, IBA).

#### 3.8.3. Excised Cucumber (*Cucumis sativus* L.) Cotyledon Root Induction for Auxin-like Effect

Commercial seeds of cucumber (*Cucumis sativus* L.) were used to evaluate the stimulant effect on germination, according to the method described by Zhao et al. (1992) [48]. Cucumber seeds were placed in plastic trays with 0.7% agar solidified Knop nutrient medium that consist of 0.8 g/L of Ca(NO_3_)_2_, 0.2 g/L of KNO_3_, 0.2 g/L of KH_2_PO_4_ and 0.2 g/L of Mg_2_SO_4_·7 H_2_0 and then transferred to an incubator maintained at 24 °C for 3–5 days in darkness. Cotyledons with small (1–2 mm) hypocotyl section were excised from seeds and transferred to Petri dishes of 60 mm diameter (five per Petri dish) containing filter paper moistened with 3 mL distilled water (negative control), commercial algae product (positive control) and algal suspension (at concentrations of 0.5 and 2 g/L). The number of roots formed at the bases of 5 cotyledons was counted. The mean number of roots, from each treatment, was compared to the controls and evaluated using a standard curve for comparison made by a specific auxin (Indol-3-butyric acid, IBA).

#### 3.8.4. Excised Cucumber (*Cucumis sativus*, L.) Expansion Test for Cytokinin-like Effect

Commercial seeds of cucumber (*Cucumis sativus* L.) were used to evaluate the stimulant effect on growth according Zhao et al.’s (1992) [48] protocol. Seeds grown were carried out as explained in the Section 3.8.3. (incubator at 24 °C for 3–5 days in darkness). After the incubation period of the cucumber seeds, five uniform cotyledons were transferred to Petri dishes of 60 mm in diameter, which contained the wet filter paper with 3 mL of distilled water (negative control), commercial algae product (positive control) and algal suspension obtained by each treatment at a final concentration of 0.5 and 2 g/L. There should be four replicates (petri dishes) per treatment. The weight of the control and the four replicated was measured and evaluated using a standard curve for comparison made by a logarithmic concentration range of a specific cytokinin (6-Benzylaminopurine, BAP).

### 3.9. Statistical Analyses

Statistical data analyses were performed using the Statgraphics Centurion XVII software package. Data, in percentage, were arcsin(x^1/2^) transformed. Multifactor ANOVA tests were used to study the effect of the factors (cell disruption, enzymatic hydrolysis, centrifugation and algal extract concentration) and their interactions at a 95% confidence level for all the protocols were tested.

## 4. Conclusions

The good results of brewery wastewater based *Scenedesmus obliquus* treatment were evidenced in this work by the fact that the obtained biomass is useful as a biostimulant for seeds of watercress, mung bean and cucumber, which are agricultural models.

Different downstream processes were applied to evaluate the final bioactivity potential of the microalgal biomass as a biostimulant. It should be highlighted the 140% obtained for the Germination Index (GI; control = 100%) by T1 treatment—without any treatment (centrifugation, cell disruption nor enzymatic hydrolysis), at 0.1 g/L concentration. For auxin-like mung bean root formation the best achieved treatment was T8 (with cell disruption, enzymatic hydrolysis and centrifugation at 0.5 g/L; 167.9%) and for cucumber, the best ones were T3 (without cell disruption, with enzymatic hydrolysis and no centrifugation) and T7 (with cell disruption, enzymatic hydrolysis and no centrifugation), both at 0.5 g/L (141.9%). The best results for cytokinin-like on cucumber root expansion were achieved by T4 (without cell disruption, with enzymatic hydrolysis and centrifugation at 2 g/L; 287.5%), followed by T6 (with cell disruption, without enzymatic hydrolysis and with centrifugation) at 0.5 g/L (245%).

Except for the cytokinin-like effect, T1 achieved good results for all bioassays, which is very advantageous as there is no energy, chemicals and time consumption, resulting in lower costs and better profitability of the product. 

Multifactor ANOVA statistical analysis revealed that, in general, the main factor affecting the study parameters is the enzymatic hydrolysis and the interaction of extract concentration-enzymatic hydrolysis, being much more evident on the germination index of the seeds.

These results clearly demonstrated the potential of the *S. obliquus*, after being cultivated and removing the nutrients from the brewery wastewater, to be used in a more sustainable agriculture, avoiding the use of chemicals, saving energy and GHG emissions. Nevertheless, large research is still needed, in order to optimize the best treatments and conditions, as well as the understanding of the bioactive activity of the microalga biomass/extract and its effect on germination and growth of roots and cotyledons. Finally, wariness should be paid on the eventual presence of heavy metals, toxicity and pathogens in order to access the impact of the biostimulant product developed on a sustainable and healthy agricultural practice.

The wastewater treatment-based microalgae and further use of the biomass on sustainable agriculture should be implemented in the near future in a circular bioeconomy platform.

## Figures and Tables

**Table 1:** Results of the four bioassays applied on the microalgal biomass from *Scenedesmus obliquus* for the eight treatments (T1–T8) tested at two different concentrations (in g/L). The gibberellin-like effect was determined through a germination index bioassay in watercress seeds (*Lepidium sativum*), the auxin-like effect was determined according to the mung bean (*Vigna radiata*) and cucumber (*Cucumis sativus*) cotyledon root formation bioassay and the cytokinin-like effect according to the excised cucumber (*Cucumis sativus*) cotyledon root expansion bioassay. Results are expressed in a percentage as means ± standard deviation (*n* = 3) considering distilled water as the control (100%). The values highlighted in bold represent the two best results for each bioassay for both microalgal concentration.

	Gibberellin-Like Effect		Auxin-Like Effect				Cytokinin-Like Effect	
	Germination Index (Watercress Seeds)		Mung bean Bioassay (Root Formation)		Cucumber Bioassay (Root Formation)		Cucumber Expansion Bioassay (Mass of Cotyledons)	
Treatments								
	0.1 g/L	0.5 g/L	0.5 g/L	2 g/L	0.5 g/L	2 g/L	0.5 g/L	2 g/L
Commercial	93.1 ± 2.8	102.1 ± 3.2		94.3 ± 13.1		125.8 ± 12.1		92.9 ± 13.1
T 1	**139.1 ± 1.9**	**118.9 ± 3.2**	137.7 ± 3.3	109.4 ± 26.7	130.6 ± 21.3	122.6 ± 21.9	58.8 ± 8.9	68.7 ± 9.9
T 2	118.8 ± 3.1	108.2 ± 4.5	137.7 ± 3.3	147.2 ± 27.9	134.7 ± 28.4	123.4 ± 11.6	**160.9 ± 68.7**	71.9 ± 11.1
T 3	86.5 ± 5.2	88.1 ± 5.8	126.4 ± 4.3	37.7 ± 10.1	**141.9 ± 4.6**	123.4 ± 11.6	68.5 ± 7.3	**200.3 ± 38.8**
T 4	81.1 ± 4.9	91.1 ± 6.2	113.2 ± 22.6	122.6 ± 15.1	99.2 ± 15.4	112.1 ± 10.6	89.6 ± 24.3	**287.5 ± 59.2**
T 5	109.2 ± 3.2	**113.1 ± 4.9**	145.3 ± 11.8	**164.2 ± 8.1**	101.6 ± 22.7	101.6 ± 12.5	88.7 ± 29.9	70.8 ± 11.1
T 6	**121.8 ± 5.8**	100.2 ± 2.3	**150.9 ± 8.7**	**158.5 ± 9.8**	122.6 ± 8.7	100.8 ± 18.3	**245.1 ± 55.9**	65.1 ± 21.8
T 7	97.9 ± 6.1	84.6 ± 3.8	118.9 ± 16.9	75.5 ± 7.8	**141.9 ± 4.6**	**127.4 ± 18.3**	100.99 ± 32.5	80.8 ± 20.4
T 8	79.3 ± 5.4	82.8 ± 4.1	**167.9 ± 14.2**	135.8 ± 16.9	112.1 ± 17.7	**140.3 ± 14.3**	93.9 ± 12.5	68.3 ± 20.1

**Table 2:** Multifactor ANOVA testing the effect of the microalgal extract concentration, the cell disruption, enzymatic hydrolysis and centrifugation on the germination index of the watercress seeds (*Lepidium sativum*), the presence of auxins (root development with *Vigna radiata* and cotyledon root development with *Cucumis sativus*) and the presence of cytokinins (cotyledon root expansion with *Cucumis sativus* seeds; from Table 1 results). The data variability is attributable to the main effect of each factor and the interaction found, as indicated by the *p*-value. The contribution of each factor was expressed as the percentage variation of the response (F ratio of each factor relative to the sum of all F- ratios).

Response	Statistics	Algal Concentration	Cell Disruption	Enzymatic Hydrolysis	Centrifugation	Concentration-Enzymatic Hydrolysis
**Germination index**	Variation (%)	0.91	0.99	59.9	1.6	36.58
(*Lepidium sativum*)	*p*-value	0.7214	0.5528	0.0001	0.455	0.0003
**Root development**	Variation (%)	39.94	27.12	25.1	7.8	14.19
(*Vigna radiata*)	*p*-value	0.027	0.1236	0.138	0.3395	0.2974
**Cotyledon root formation**	Variation (%)	42.54	37.9	6.45	6.65	6.45
(*Cucumis sativus*)	*p*-value	0.1213	0.18	0.5746	0.5672	0.8102
**Cotyledon root expansion**	Variation (%)	0.78	0.52	25.33	9.66	63.7
(*Cucumis sativus*)	*p*-value	0.9917	0.8966	0.3319	0.5454	0.0894

**Table 3:** Brewery wastewater composition in terms of pH [5].

Pollutant	pH	NH_3_ (mg·L^−1^)	TKN (mg·L^−1^)	PO_4_^3-^ (mg·L^−1^)	P-PO_4_^3-^ (mg·L^−1^)	COD (mg·O_2_·L^−1^)
Composition	7.2	29.4 ± 1.4	47.6 ± 2.8	44.0 ± 0.1	14.22 ± 0.08	295.7 ± 0.0
